# Unexpected Attraction of Polarotactic Water-Leaving Insects to Matt Black Car Surfaces: Mattness of Paintwork Cannot Eliminate the Polarized Light Pollution of Black Cars

**DOI:** 10.1371/journal.pone.0103339

**Published:** 2014-07-30

**Authors:** Miklos Blaho, Tamas Herczeg, Gyorgy Kriska, Adam Egri, Denes Szaz, Alexandra Farkas, Nikolett Tarjanyi, Laszlo Czinke, Andras Barta, Gabor Horvath

**Affiliations:** 1 Environmental Optics Laboratory, Department of Biological Physics, Physical Institute, Eötvös University, Budapest, Hungary; 2 Group for Methodology in Biology Teaching, Biological Institute, Eötvös University, Budapest, Hungary; 3 Danube Research Institute, Centre for Ecological Research, Hungarian Academy of Sciences, Budapest, Hungary; Washington State University, United States of America

## Abstract

The horizontally polarizing surface parts of shiny black cars (the reflection-polarization characteristics of which are similar to those of water surfaces) attract water-leaving polarotactic insects. Thus, shiny black cars are typical sources of polarized light pollution endangering water-leaving insects. A new fashion fad is to make car-bodies matt black or grey. Since rough (matt) surfaces depolarize the reflected light, one of the ways of reducing polarized light pollution is to make matt the concerned surface. Consequently, matt black/grey cars may not induce polarized light pollution, which would be an advantageous feature for environmental protection. To test this idea, we performed field experiments with horizontal shiny and matt black car-body surfaces laid on the ground. Using imaging polarimetry, in multiple-choice field experiments we investigated the attractiveness of these test surfaces to various water-leaving polarotactic insects and obtained the following results: (i) The attractiveness of black car-bodies to polarotactic insects depends in complex manner on the surface roughness (shiny, matt) and species (mayflies, dolichopodids, tabanids). (ii) Non-expectedly, the matt dark grey car finish is much more attractive to mayflies (being endangered and protected in many countries) than matt black finish. (iii) The polarized light pollution of shiny black cars usually cannot be reduced with the use of matt painting. On the basis of these, our two novel findings are that (a) matt car-paints are highly polarization reflecting, and (b) these matt paints are not suitable to repel polarotactic insects. Hence, the recent technology used to make matt the car-bodies cannot eliminate or even can enhance the attractiveness of black/grey cars to water-leaving insects. Thus, changing shiny black car painting to matt one is a disadvantageous fashion fad concerning the reduction of polarized light pollution of black vehicles.

## Introduction

Nowadays cars have frequently a matt black or dark grey finish. Matt car surfaces can be produced via paint or foil. Such a finishing gives an eye-striking, unusual matt colour to the car-body. Since this painting or covering is rather expensive, predominantly the luxurious cars are produced to be matt.

**Figure 1:**
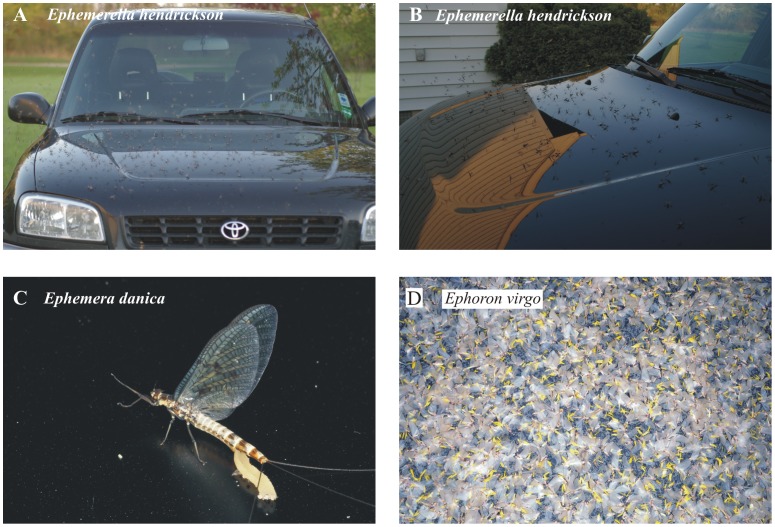
Mayflies attracted *en masse* to shiny black cars due to the highly and horizontally polarized light reflected from the car-body. (A, B) Mass-swarming *Ephemerella hendrickson*. (C) Egg-laying *Ephemera danica*. (D) Thousands of mass-swarming female *Ephoron virgo* mayflies landed on a windscreen, onto which they laid their yellow egg batches. Photos A and B were taken by Dr. Rebecca Allen (Michigan State University, USA), while photos C and D originate from Dr. György Kriska (Eötvös University, Budapest, Hungary).

Shiny car-bodies attract water-leaving insects [1]–[3], because the hood, roof and boot reflect horizontally polarized light [4], [5], and these insects are lured to this optical signal since they detect water by means of the horizontal polarization of water-reflected light [6]–[19]. This positive polarotaxis induced by the reflection polarization of artificial surfaces is the main reason for polarized light pollution [20]. Thus, shiny black cars are typical sources of polarized light pollution [4], [21], a spectacular consequence of which can be seen in Fig. 1 showing mass-swarming mayflies attracted to shiny black cars. The mayflies in Fig. 1 laid their egg batches (each containing 6000-9000 eggs) onto car-bodies, and these eggs perish quickly due to dehydration. *Ephoron virgo* (Ephemeroptera: Polymitarcyidae; Fig. 1D) is not only endangered [22], but also a highly protected mayfly species in Europe [23].

**Figure 2:**
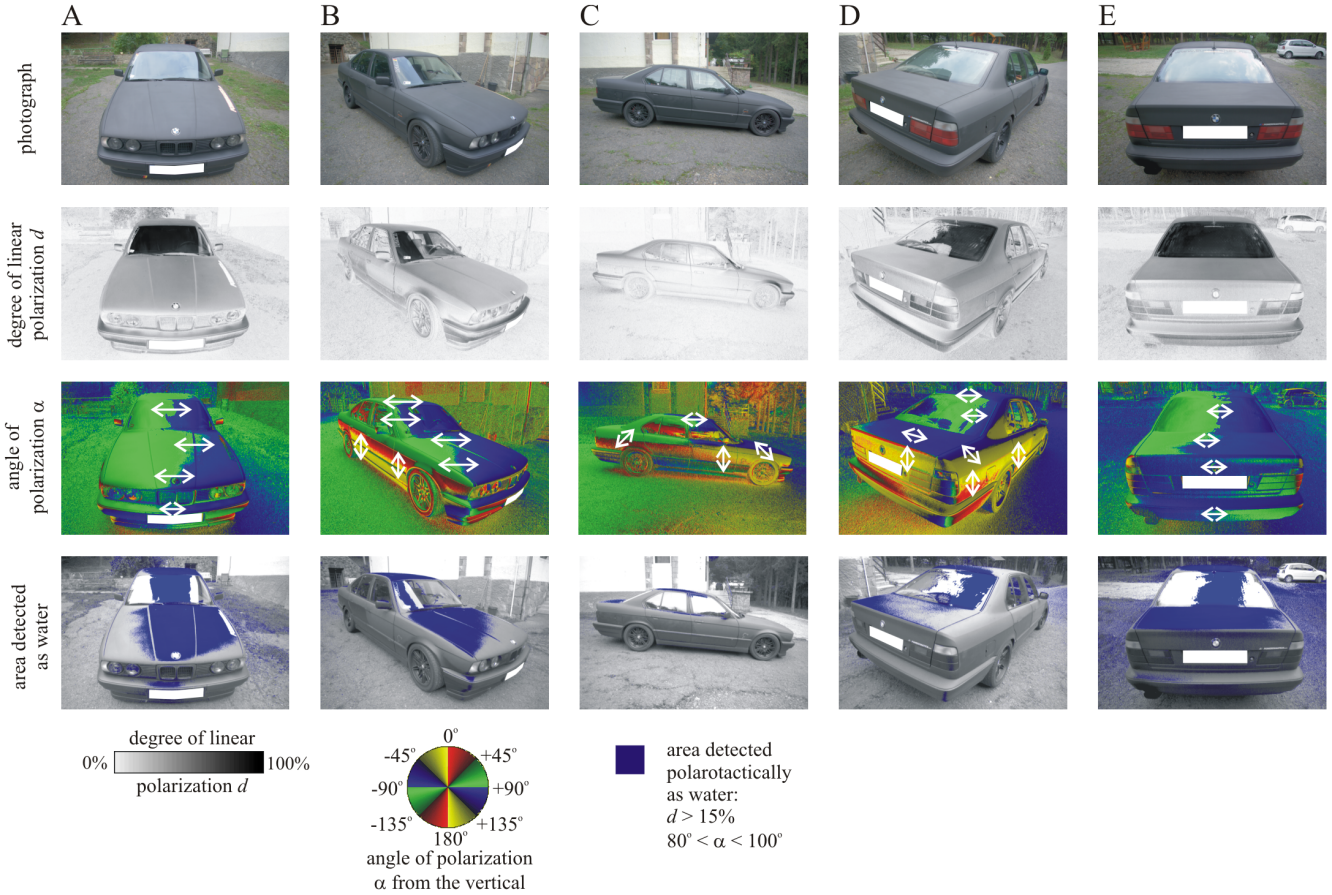
Photograph, patterns of the degree of linear polarization *d* and the angle of polarization α (clockwise from the vertical), and areas detected as water by polarotactic insects (for which the reflected light has the following characteristics: *d*>15%, 80^o^<α<100^o^) of a matt black car measured with imaging polarimetry from five different directions of view in the blue (450 nm) part of the spectrum. The angle of elevation of the optical axis of the polarimeter was −20^o^ from the horizontal. The number-plate of the car is screened by a white rectangle.

A new fashion fad is that cars are painted matt black or matt dark grey (row 1 in Fig. 2, Fig. S1A-E). Alternatively, the whole car-body or its fragments (e.g., the roof, hood or boot) are covered with carbon foil resulting in a similarly matt black/grey appearance (Fig. S1F). Since rough (matt) surfaces depolarize the reflected light [4], [5], [8], [11], [14], [16]–[18], [20], one of the possible ways of elimination of polarized light pollution of shiny black artificial surfaces is to make them matt [15], [20]. Thus, one could hypothesize that matt black/grey cars may less induce polarized light pollution, because they may reflect weakly polarized light, that does not attract polarotactic insects. If this were the case, the spreading of matt black cars would be a fashion fad that could be welcomed, because they would not attract polarotactic insects, the egg batches of which would inevitably perish due to dehydration when laid onto car-bodies.

To compare the attractivenes of matt and shiny black car surfaces to polarotactic insects, we performed two field experiments with horizontal shiny and matt black/grey car-body fragments. We checked the attractiveness of these test surfaces to polarotactic mayflies, dolichopodid flies and tabanid flies. The studied mayfly species are endangered in Europe. Mayflies and dolichopodids were abundant in the site of our first experiment. Since tabanid flies are serious pests, they are neither rare nor protected species. The investigated mayflies, dolichopodids and tabanids were used simply as indicators of strongly and horizontally polarized reflected light, because they are positively polarotactic insects [15], [16], [18], [24]–[31]. We showed that changing shiny black car paintwork to matt one can be a disadvantageous fashion fad concerning the polarized light pollution of black vehicles.

## Results

### Reflection-polarization characteristics of matt black cars

Figure 2 shows the reflection-polarization characteristics of a matt black car measured from five different directions of view in the blue (450 nm) part of the spectrum with imaging polarimetry. The degree of polarization *d* of light reflected from the skylit part of the tilted windscreen was very high (85%<*d*<100%), while the light reflected from the vertical windows was only weakly polarized (*d*<15%). The roof, hood and boot reflected moderately polarized light (25%<*d*<55%), and the vertical parts of the car-body reflected weakly polarized light (*d*<15%). The spatial distribution of *d* of light reflected from the roof, hood and boot was rather homogeneous. The tilted windscreen and the horizontal roof, hood and boot reflected horizontally polarized light, while the other (vertical and tilted) parts of the car-body reflected light with vertical or oblique directions of polarization. Areas of the car-body that reflect light with degree of polarization high enough (*d*>15%) and with nearly horizontal direction of polarization (80^o^<α<100^o^) are sensed as water by polarotactic insects (Horváth and Varjú 2004; Kriska *et al*. 2009). According to Fig. 2, these areas of the matt black car-body occured on the windscreen, roof, hood and boot. The reflection-polarization characteristics of car-bodies covered by matt black/grey carbone foils are quite similar (Fig. S5).

### Reflection-polarization characteristics of test surfaces

**Figure 3:**
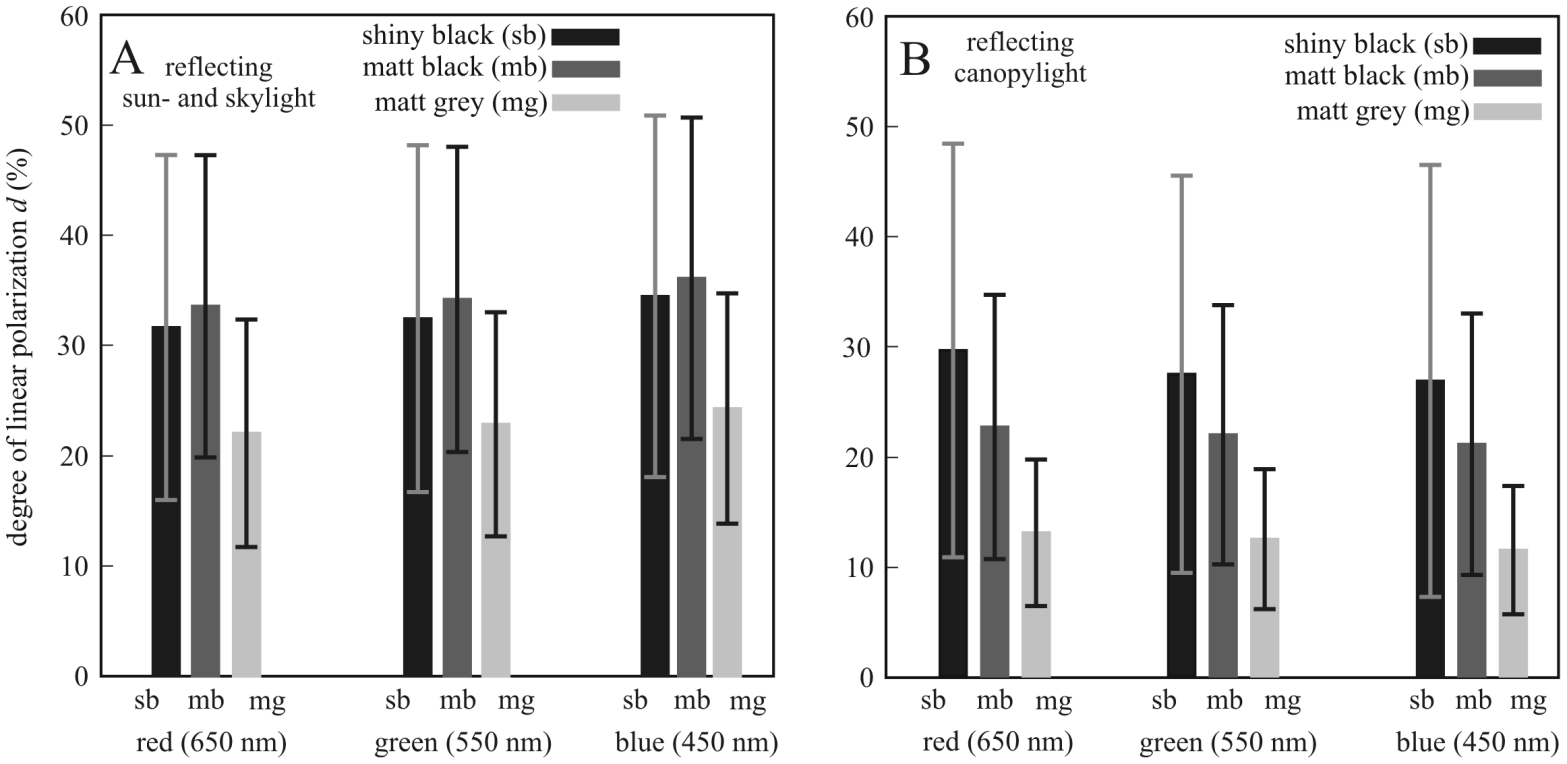
Degree of linear polarization *d* of the shiny black (sb), matt black (mb) and matt grey (mg) horizontal test surfaces used in experiments 1 and 2 measured with imaging polarimetry in the red (650 nm), green (550 nm) and blue (450 nm) parts of the spectrum when sun- and skylight (A, Supplementary Fig. S4I) or canopylight originating from trees and bushes (B, Supplementary Fig. S4II) was reflected by the test surfaces. Columns: averages. Vertical bars: standard deviations. The average is calculated for the whole area of each test surface (corresponding to 2500×4000 = 10 000 000 pixels in the pictures and polarization patterns).

Figure 3 shows and Table 1 contains the average and standard deviation of the degree of polarization *d* of light reflected from the three horizontal test surfaces (shiny black, matt black, matt grey) used in experiments 1 and 2 measured with imaging polarimetry in the red, green and blue parts of the spectrum when the surfaces reflected sun- and skylight, or light from trees and bushes (Fig. S4). The major part of the test surfaces reflected horizontally polarized light, a small part reflected vertically or obliquely polarized light, while from a neutral point (at the border of the horizontally and vertically polarizing surface regions) unpolarized light was reflected. The shiny black and matt black test surfaces reflected light with the highest *d*, while the matt grey surface was the weakest polarizing (Table 1, Fig. 3). The polarization characteristics of the black and grey test surfaces depended only slightly on the wavelength. When the test surfaces reflected sun- and skylight, their degree of polarization was higher in comparison to the situation when they reflected light from trees and bushes. In the blue spectral range the least standard deviation of *d* occurred on the matt grey test surface, and in the green part of the spectrum the least standard deviation of *d* possessed the matt grey test surface (Table 1). Comparing the reflection-polarization characteristics of shiny/matt, black/grey car-bodies (Fig. 2, Fig. S5) with those of our test surfaces (Fig. 3, Fig. S4, Table 1), it was evident that they were practically the same. Thus, our test surfaces imitated well the polarization characteristics of matt/shiny and black/grey car-bodies.

**Table 1:** Degree of linear polarization *d* (average ± standard deviation, averaged to the whole surface area) of the three horizontal test surfaces (shiny black, matt black, matt grey) used in experiments 1 and 2 measured with imaging polarimetry in the red (650 nm), green (550 nm) and blue (450 nm) parts of the spectrum when sun- and skylight (Fig. S4I) or canopylight originating from trees and bushes (Fig. S4II) was reflected by the test surfaces.

light incident to the test surfaces	test surface	degree of linear polarization *d* (%)		
		red	green	blue
**sun- skylight**	**shiny black**	31.6±15.7	32.4±15.7	34.5±16.4
	**matt black**	33.6±13.7	34.2±13.9	36.1±14.6
	**matt grey**	22.0±10.3	22.9±10.2	24.3±10.5
**canopylight from trees and bushes**	**shiny black**	29.6±18.8	27.4±18.0	26.8±19.6
	**matt black**	22.6±12.0	21.9±11.8	21.1±11.9
	**matt grey**	13.0±6.7	12.4±6.4	11.4±5.8

According to Table 1 (see also Fig. 3), depending on the direction of view, in the blue, green and red spectral ranges the standard deviation Δ*d* of *d* of the shiny black test surface (blue: ±16.4–19.6%, green: ±15.7–18.0%, red: ±15.7–18.8%) was 1.9–2.8, 1.8–2.5 and 1.5–2.8 times higher, respectively, than that of the matt grey surface (blue: ±5.8–10.5%, green: ±6.4–10.2%, red: ±6.7–10.3%). Similarly, Δ*d* of the matt black test surface (blue: ±11.9–14.6%, green: ±11.8–13.9%, red: ±12.0–13.7%) was 1.4–2.1, 1.4–1.8 and 1.3–1.8 times higher, respectively, than that of the matt grey surface. These results are important in the explanation of our finding that the attractiveness of the horizontally polarizing matt grey car-body fragment to mayflies was significantly larger than that of the two black test surfaces (see Discussion).

### Attractiveness of test surfaces to mayflies, dolichopodids and tabanids

**Figure 4:**
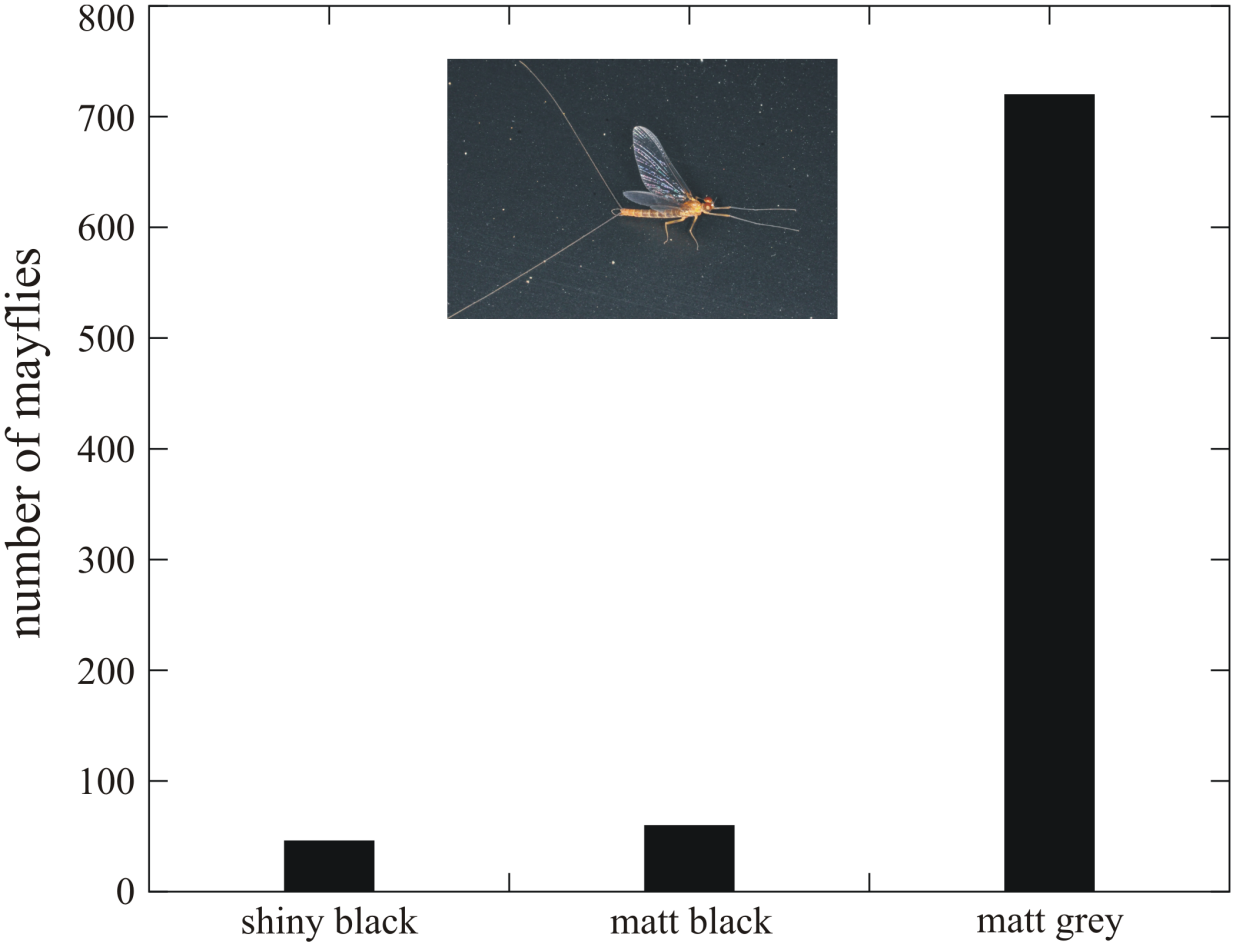
Total numbers of mayflies landed on the shiny black, matt black and matt grey horizontal test surfaces in experiment 1. The inset is a photograph of a mayfly landed on the shiny black test surface. The number of repetition is 6 (see Materials and methods, and Discussion).

Figure 4 shows the numbers of mayflies landed on the three test surfaces in experiment 1 (Table S1). There was no statistically significant difference between the attractiveness of the shiny black and matt black test surfaces to mayflies (Table 2). Interestingly, the matt grey test surface attracted about 10.7–15.7 times more mayflies than the two black test surfaces, which differences were statistically significant (Table 2). This result was very surprising, and we propose an explanation for it in the Discussion.

**Table 2:** Statistical comparisons (Kruskal Wallis and Mann-Whitney U test) between the numbers of mayflies landed on the shiny black, matt black and matt grey horizontal test surfaces in experiment 1 (Fig. 4, Table S1).

comparison	test type	test result	significancy
shiny black *versus* matt black *versus* matt grey	Kruskal-Wallis	H = 176.2, df = 2, p<0.0001	significant
shiny black *versus* matt grey	Mann-Whitney U with Bonferroni correction	U = 172.5, p<0.0001	significant
shiny black *versus* matt black	Mann-Whitney U with Bonferroni correction	U = 4372.5, p = 0.38	not significant
matt black *versus* matt grey	Mann-Whitney U with Bonferroni correction	U = 210, p<0.0001	significant

**Figure 5:**
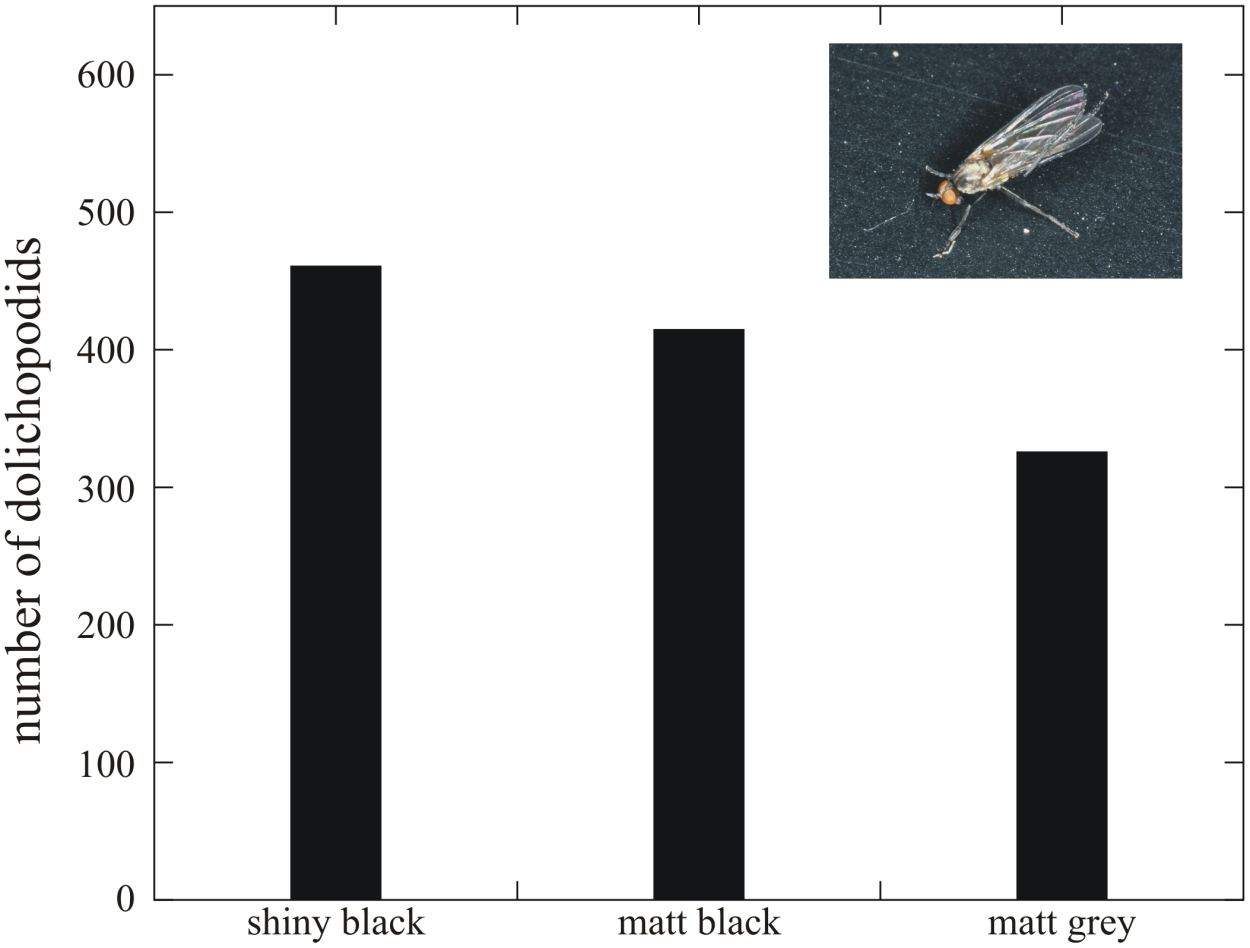
Total numbers of dolichopodids landed on the shiny black, matt black and matt grey horizontal test surfaces in experiment 1. The inset is a photograph of a dolichopodid fly landed on the matt black test surface. The number of repetition is 6 (see Materials and methods, and Discussion).

Figure 5 shows the numbers of dolichopodids landed on the three test surfaces in experiment 1 (Table S1). The shiny black test surface was the most attractive to dolichopodids and the matt grey surface was the least attractive. The differences between the attractiveness of the shiny black and matt grey as well as between the matt black and matt grey test surfaces were significant, while there was no significant difference between the numbers of dolichopodids landed on the shiny black and matt black surfaces (Table 3).

**Table 3:** Statistical comparisons (Kruskal Wallis and Mann-Whitney U test) between the numbers of dolichopodids landed on the shiny black, matt black and matt grey horizontal test surfaces in experiment 1 (Fig. 4, Table S1).

comparison	test type	test result	significancy
shiny black *versus* matt black *versus* matt grey	Kruskal-Wallis	H = 16.8, df = 2, p = 0.0002	significant
shiny black *versus* matt grey	Mann-Whitney U with Bonferroni correction	U = 3111.5, p = 0.0001	significant
shiny black *versus* matt black	Mann-Whitney U with Bonferroni correction	U = 4166, p = 0.25	not significant
matt black *versus* matt grey	Mann-Whitney U with Bonferroni correction	U = 3468, p = 0.003	significant

**Figure 6:**
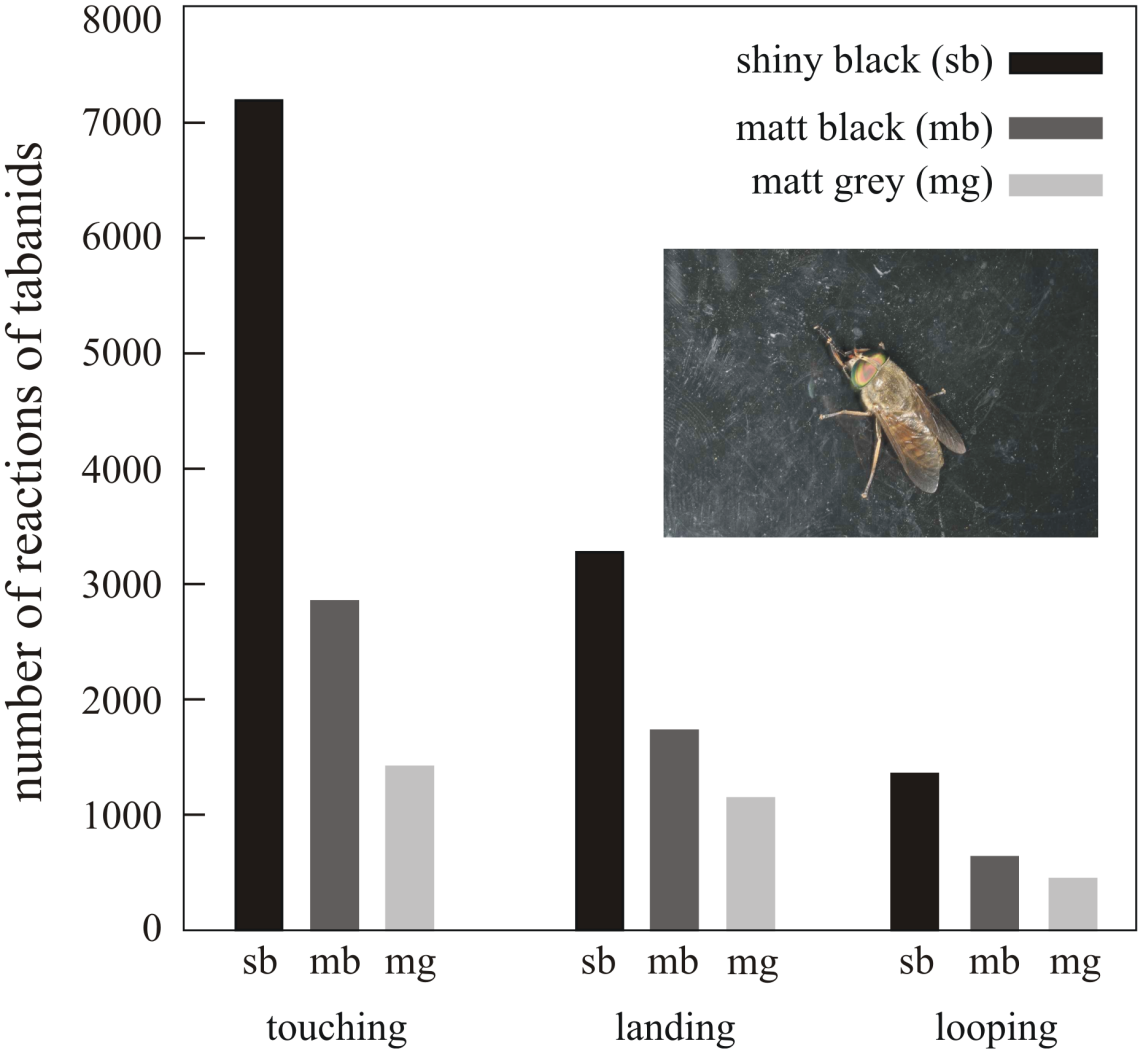
Total numbers of reactions (touching, landing and aerial looping) of tabanids to the shiny black (sb), matt black (mb) and matt grey (mg) horizontal test surfaces in experiment 2. The inset is a photograph of a tabanid fly landed on the matt grey test surface. The number of repetition is 20 (see Materials and methods, and Discussion).

Figure 6 shows the numbers of landing, touching and looping of tabanids at the three test surfaces in experiment 2 (Table S2). Considering these three reactions, the shiny black test surface was significantly the most attractive to tabanids, while considering landing and touching, the matt grey surface was significantly the least attractive. The attractiveness of the matt black test surface was between that of the shiny black and the matt grey surfaces (Fig. 6, Table 4).

**Table 4:** Statistical comparisons (Kruskal Wallis and Mann-Whitney U test) between the numbers of the three reactions (landing, touching, looping) of tabanids to the shiny black, matt black and matt grey horizontal test surfaces in experiment 2 (Fig. 6, Table S2).

comparison		test type	test result	significancy
landing	shiny black *versus* matt black *versus* matt grey	Kruskal-Wallis	H = 38.6, df = 2, p<0.0001	significant
	shiny black *versus* matt grey	Mann-Whitney U with Bonferroni correction	U = 11, p<0.0001	significant
	shiny black *versus* matt black	Mann-Whitney U with Bonferroni correction	U = 27.5, p<0.0001	significant
	matt black *versus* matt grey	Mann-Whitney U with Bonferroni correction	U = 67.5, p = 0.0003	significant
touching	shiny black *versus* matt black *versus* matt grey	Kruskal-Wallis	H = 34.2, df = 2, p<0.0001	significant
	shiny black *versus* matt grey	Mann-Whitney U with Bonferroni correction	U = 20, p<0.0001	significant
	shiny black *versus* matt black	Mann-Whitney U with Bonferroni correction	U = 44, p<0.0001	significant
	matt black *versus* matt grey	Mann-Whitney U with Bonferroni correction	U = 71.5, p = 0.0005	significant
looping	shiny black *versus* matt black *versus* matt grey	Kruskal-Wallis	H = 11.2, df = 2, P = 0.004	significant
	shiny black *versus* matt grey	Mann-Whitney U with Bonferroni correction	U = 82, P = 0.001	significant
	shiny black *versus* matt black	Mann-Whitney U with Bonferroni correction	U = 127.5, p = 0.049	not significant
	matt black *versus* matt grey	Mann-Whitney U with Bonferroni correction	U = 139.5, p = 0.10	not significant

The results of our experiments show the following: (i) The attractiveness of black car-bodies to polarotactic insects depends in complex manner on the surface roughness (shiny, matt) and species (mayflies, dolichopodids, tabanids). (ii) Depending on species, matt black car-bodies can be less or equally attractive to polarotactic insects than shiny black ones. (iii) The polarized light pollution of shiny black cars usually cannot be reduced with the use of matt painting. (iv) Non-expectedly, the matt dark grey car finish is much more attractive to mayflies (being endangered and protected in many countries) than matt black finish.

## Discussion

One could assume that changing the shiny paintwork of black car-bodies to a matt one, the polarized light pollution of the horizontally polarizing roof, hood and boot can be reduced, since roughness (mattness) depolarizes the reflected light. In our field experiments we obtained that this is not what always happens, because the investigated horizontally polarizing matt black and matt grey car-body fragments were similarly attractive, or even more attractive to polarotactic mayflies than the studied horizontally polarizing shiny black car-body fragment. The reason for this is that although (i) the matt grey car-body surface reflected light with lower degrees of polarization *d* than the shiny black surface, and (ii) the matt black car finish was less polarizing than the shiny black finish when reflecting canopylight (Table 1, Fig. 3B), this reduction of *d* was not large enough to make them unattractive to polarotactic insects. On the other hand, (iii) the matt black finish was even more polarizing than the shiny black finish when reflecting sun- and skylight (Table 1, Fig. 3A). One of the prerequisites of attraction of water-leaving insects by polarizing reflectors is that *d* of reflected light must be higher than the threshold d* of insect polarization sensitivity. Species-specific values of d* are known for certain dragonfly, mayfly and tabanid fly species [25]. Thus, the attractiveness of a car paintwork to polarotactic insects can be ensured only if *d* of reflected light is higher than d*.

More remarkably, the attractiveness of the horizontal matt grey car-body fragment to polarotactic mayflies was significantly larger than that of the two black test surfaces. One of the reasons for this could be that the standard deviation Δ*d* of *d* of the shiny black test surface was about 2–3 times higher than that of the matt grey surface, and Δ*d* of the matt black test surface was 1.3–2.1 times higher than that of the matt grey surface (Table 1, Fig. 3). Calm water bodies with a smooth surface are characterized by small Δ*d* of reflected light due to the lack of ripples [11], [32], [33]. Turbulent waters with rough, ripplening/undulating surfaces reflect light with large Δ*d*, because *d* of reflected light depends strongly on the direction of incidence relative to the normal vector of the surface, which changes randomly spatio-temporally if the water is turbulently flowing. Since the mayflies investigated in experiment 1 prefer quiescent water bodies [34], the matt grey test surface with small Δ*d* of reflected light might have imitated a calm water surface to them in the visible spectral range, while the two black test surfaces with approximately 1.5–3 times larger Δ*d* might have been sensed by mayflies as turbulent waters. Therefore, the matt grey test surface was much more attractive to mayflies than the two black test surfaces.

Another reason for the greater attractiveness of the matt grey car-body surface to mayflies can be that the matt grey test surface (with a greyness of 90%) was by 10% brighter than the black test surfaces (with a greyness of 100%), and thus the former was more attractive to mayflies due to their possible positive phototaxis. However, it has been shown earlier that the studied polarotactic mayfly species (*Baetis rhodani*, *Epeorus sylvicola*, *Rhithrogena semicolorata*) are not phototactic [24]: their attractiveness to shiny/matt white/grey/black horizontal surfaces is governed by the linear polarization of reflected light, rather than by the light intensity (brighness/darkness).

Table 1 (see also Fig. 3) provides the values of the degree of polarization *d* in three (red, green, blue) spectral ranges to show that the reflection-polarization characteristics of our test surfaces used in experiments 1 and 2 were practically independent of the wavelength in the visible part of the spectrum: at a given test surface under a given illumination condition the spectral differences in *d* are less than 5%, which is below the polarization sensitivity threshold of any known animal [35].

In our experiments mayflies, dolichopodids and tabanids attracted by test surfaces with different reflection-polarization characteristics were counted. Experiment 1 was repeated 6 times on 6 days during the extremely short (a few days) swarming period of the investigated mayflies. This meant 6×2 = 12 h observation time, during which 96 photographs were taken about each test surface (Table S1). After each photography the three test surfaces were reordered with cyclical permutation to eliminate site effects. The studied mayflies live as adult only for one day. Thus, during a given 2-hour session of experiment 1 (on a given day) a given mayfly might react to certain test surfaces more than once, meaning pseudo-replication of this experiment after the reordering of the test surfaces every 5 minutes (24 times in the 2 h of a given session). Such a possible pseudo-replication could not be avoided during the 2 h of a given session, because the test surfaces must have been dry. Pseudo-replication could have been avoided only with the use of sticky test surfaces. However, the matt black test surfaces must not be covered with any glue, since the glue layer would make them shiny. On the other hand, each day of the 6-day experiment 1 always new mayflies might have occurred due to their one-day life-time. Thus, pseudo-replication might have been minimal in experiment 1. The adults of dolichopodids live more than one day, thus, considering the numbers of dolichopodids, some degree of pseudo-replication may not be excluded in experiment 1. However, it is highly probable that every day of the 6-day experiment 1 not the same dolichopodid individuals reacted to our test surfaces all the time. Similar was true for the number of tabanids in the 20-day experiment 2 repeated 20 times with hourly cyclically permutated re-ordering of the three test surfaces. According to our results and experiences gained in our earlier similar field experiments with mayflies, dolichopodids and tabanids [14], [16], [18], [24]–[31], the number of repetitions and the duration of experiments 1 and 2 were large enough to detect significant differences in the attractiveness of test surfaces to polarotactic insects.

In our research the exact species names are practically irrelevant, since the studied water-leaving insects were only indicators of the polarized light pollution of the investigated test surfaces. The only important aspect was that positively polarotactic insects should be involved into our field experiments, the aim of which was to test the attractiveness of matt/shiny and black/grey car-bodies. All the involved species are positively polarotactic as shown earlier.

In every year more and more cars are running on the roads. They are predominantly shiny, and if their paint strongly absorbs light in a given spectral range, they induce strong polarized light pollution [4], [21] like the black or dark grey asphalt roads themselves [20], [24], [36]. We showed here that, unfortunately, this kind of light pollution cannot be eliminated by the use of matt black/grey car paintworks available presently in the market. The technology of matt paintworks has been developed to provide car-owners with a striking visual appearance of their cars and/or the protection of vulnerable paintworks against scratches. It would be worth improving this technology to ensure a much greater reduction of reflection polarization in order to eliminate polarized light pollution of car-bodies. If this were realized, the mattness of black cars could be an advantageous fashion fad considering the protection of endangered populations of polarotactic water-leaving insects.

The physical reason for the high polarization reflection (eliciting attractiveness to polarotactic insects) of black car surfaces is the following: A shiny (smooth) surface of a given dielectric medium reflects two components. The first component, the light reflected from the air-medium interface, is partially linearly polarized with a direction of polarization parallel to the surface. The second component, the light backscattered from the medium and refracted at the medium-air interface, is also partially linearly polarized but with direction of polarization perpendicular to the surface. The superposition of these orthogonally polarized components reduces the net degree of polarization of surface-reflected light. If the first or the second component dominates, the degree of polarization is high with a direction of polarization parallel or perpendicular to the surface, respectively. In the case of the smooth (shiny) surface of a black medium the first component dominates, because the second component is strongly absorbed by the black medium, thus, the degree of polarization of reflected light is high. If the surface of a black medium is rough (matt), in a microscopic scale it is composed of countless tiny surface fragments (facets), the surfaces of which are smooth on their own but oriented randomly in all possible directions. Due to these random facet orientation the surface reflects light diffusely (in all possible directions). A given facet reflects light with high degrees of polarization due to the absorption of the above-mentioned second component, but the direction of polarization of facet-reflected light is random because of the random facet orientation. Furthermore, incident light can also be reflected more than once from different facets. All these result in that the net degree of polarization of light reflected by a matt surface is reduced because of the superposition of the numerous individual facet-reflected components with random directions of polarization. More details of the physics/optics of absorbing and reflecting materials can be read e.g. in [37], [38].

## Materials and Methods

### Experiment 1

Experiment 1 was performed between 15 and 29 May 2013 on six warm days in the Hungarian Duna-Ipoly National Park at Dömörkapu (47^o^ 40' N, 19^o^ 03' E), where an asphalt road is running parallel (at a distance not more than 7 m) to a mountain creek. The creek is the emergence site of different mayfly (Ephemeroptera: Baetidae, Heptageniidae) and dolichopodid fly (Diptera: Dolichopodidae) species. At dusk from May to July every year adult mayflies and dolichopodids emerge from the creek and swarm in large numbers near or above the asphalt road, which is thus an ideal place for choice experiments. As the studied mayfly species are endangered in Europe, we obtained a permission from the Central Danube Environmental Protection and Water Management Inspectorate to perform our field experiment at this site. In this experiment our aim was to test the attractiveness of horizontal car-body parts, such as the hood, roof and boot of car-bodies to mayflies and dolichopodids, which are positively polarotactic insects [14], [24]–[26]. There were three different test surfaces: (i) shiny black (greyness  = 100%), (ii) matt black (greyness  = 100%) and (iii) matt grey (greyness  = 90%). They were composed of a metal plate (80 cm×80 cm, 10 kg) painted or covered with the same paints or carbon foil as used presently in the car industry. These test surfaces were produced by a professional Hungarian firm (Lakk-Mix Ltd., Budapest) dealing with car-bodies, car-body painting and covering, and using strict standardized methods. Only one of the test surfaces was covered by a matt grey carbone foil (Avery 502), the other two surfaces were painted by RAL (shiny black, matt black) paint.

These car-body fragments were laid on the asphalt surface (of a small bridge running above the creek) along a straight line 1 m apart from each other. On a given day the experiment began at 19 h ( =  local summer time  =  UTC+2 h) and stopped at 21 h. The order of the test surfaces was cyclically permutated every 5 minutes to eliminate site effects. After such a reordering, we photographed all three test surfaces with a digital camera (Nikon D90) to document the insects landed on or flying immediately (a few dm) above them (Figs. S2, S3). Later, in the laboratory we counted the number of mayflies and dolichopodids on these photographs. Although these mayflies and dolichopodids could not be taxonomically identified, they surely belonged to the order Ephemoreptera, family Baetidae, Heptageniidae and order Diptera, family Dolichopodidae, respectively, as was visually determined by one of the authors (G. K.), who is an expert of these insects. According to our earlier field experiments at the same site [24]–[26], we know that the following mayfly and dolichopodid species occurred in the air during experiment 1: *Baetis rhodani*, *Epeorus sylvicola*, *Rhithrogena semicolorata* (mayflies), *Dolichopus ungulatus*, *Dolichopus acuticornis*, *Dolichopus agilis* (dolichopodids).

### Experiment 2

Experiment 2 was performed between 24 June and 27 July 2013 twenty times on sunny, warm days on a Hungarian horse farm in Szokolya (47^o^ 52' N, 19^o^ 00' E), where tabanid flies are abundant in summer [18], [25]–[31]. Our aim was to test the attractiveness of the three (shiny black, matt black, matt grey) test surfaces used in experiment 1 to tabanids, which are positively polarotactic insects [16], [28], [31], [39]. The test surfaces were laid on the ground along a straight line 1 m apart from each other in a meadow near the horse farm, 5 m from a row of trees and bushes. Two persons sat 2 m from the row of the three test surfaces and continuously counted the reactions of tabanids to them. On a given day the experiment began at 9 h (UTC+2 h) and stopped at 14 h. The order of the test surfaces was cyclically permutated hourly to eliminate site effects. Three different tabanid reactions were distinguished: (1) aerial looping (a flying tabanid approached the test surface and performed at least one loop in the air above it at a height of a few decimeters), (2) touch-down (a tabanid touched at least once the test surface then flew away), and (3) landing (a tabanid landed on the test surface and remained on it at least for 3 seconds). These reactions are typical to tabanid flies at horizontally polarizing reflecting surfaces on the ground [16], [40]. Although the observed tabanids could not be taxonomically identified, they surely belonged to the family Tabanidae as was visually determined by the observers (authors of this work), who are experience in tabanid field experiments. In the horse farm two other experiments using different tabanid traps ran simultaneously with experiment 2. Since the tabanids captured in these experiments were later identified (by Mónika Gyurkovszky and Róbert Farkas, Department of Parasitology and Zoology, Faculty of Veterinary Science, Szent István University, Budapest), we know that the following tabanid species occurred in the air during experiment 2: *Tabanus tergestinus*, *T. bromius*, *T. bovinus*, *T. autumnalis*, *Atylotus fulvus*, *A. loewianus*, *A. rusticus*, *Haematopota italica*.

### Imaging polarimetry

The reflection-polarization characteristics of cars and the test surfaces used in experiments 1 and 2 were measured by imaging polarimetry in the red (650±40 nm  =  wavelength of maximal sensitivity ± half bandwidth of the CCD detectors of the polarimeter), green (550±40 nm) and blue (450±40 nm) spectral ranges. Mayflies, dolichopodids and tabanids possess ultraviolet-, blue- and green-sensitive photoreceptors [41]. It is, however, still unknown in which spectral range these insects sense the polarization of light. The method of imaging polarimetry has been described in detail by Horváth and Varjú [11], [32]. Here we present only the polarization patterns measured in the blue part of the spectrum. In the case of the black and grey cars and test surfaces similar patterns were obtained in the red and green spectral ranges, because due to their colourless feature their reflection-polarization characteristics depend only slightly on the wavelength of light due to the blueness of incident skylight.

### Statistical analysis

Non-parametric Kruskal-Wallis tests [42] were used to compare the reactions of attracted insects (Tables S1 and S2) to the three different test surfaces in experiments 1 and 2. Since the Kruskal-Wallis tests were significant for all three insect groups (dolichopodids, mayflies and tabanid flies), we have done separate Mann-Whitney U tests with Bonferroni correction as post-hoc comparisons [42] to find out which groups differ significantly. All statistical tests were performed with the use of the program Statistica 7.0.

### Field study permits/approvals

Many thanks to Csaba Viski (Szokolya, Hungary), who allowed our experiments on his horse farm. We are grateful to Mónika Gyurkovszky and Prof. Róbert Farkas (Department of Parasitology and Zoology, Faculty of Veterinary Science, Szent István University, Budapest) for taxonomically identifying the tabanid species occurring in the air during our experiment 2. The logistic help of István Gubek (Eötvös University, Budapest) is also acknowledged. We thank Györgyi Antoni (Center for Innovation and Grant Affairs, Eötvös University, Budapest) and Emese Kovács (Vörösmarty Tourist House, Mátraháza, Hungary) for providing us with the matt black cars in Figures 2 and S5. Thanks are also to Rebecca Allen (Michigan State University, USA) for the photos in Fig. 1A,B. We thank the permission from the Central Danube Environmental Protection and Water Management Inspectorate to investigate mayflies.

## Conclusions

From the results of our field experiments presented here we conclude that making matt the car-body cannot reduce the polarized light pollution of black cars. Matt car surfaces can even attract more individuals of certain polarotactic insect species (e.g. mayflies) than shiny black cars. Thus, changing shiny black paintwork to matt one can be a disadvantageous fashion fad concerning environmental protection.

## Supporting Information

Figure S1 **Cars with different matt black/grey painting (A-E), or carbon foil on the hood and roof (F) (photographs taken by Gábor Horváth).** The number-plates are screened by white rectangles.
(DOC)

Figure S2 **Photographs of the shiny black, matt black and matt grey horizontal car-body fragments used in experiment 1 with some mayflies and dolichopodids above or on the test surfaces.** On such photographs were counted the attracted insects.
(DOC)

Figure S3 **Photographs of mayflies (A, B), dolichopodids (C) and tabanids (D) landed on the car-body fragments used in experiments 1 and 2.** (A) An egg laying female (down) and a male (up) *Rhithrogena semicolorata* mayfly on the matt black test surface. (B) Male *R. semicolorata* on the shiny black test surface. (C) A dolichopodid fly on the matt black test surface. (D) Tabanid flies on the shiny black test surface.
(DOC)

Figure S4 **Photograph, patterns of the degree of linear polarization** ***d*** **and the angle of polarization α (clockwise from the vertical), and areas detected as water by polarotactic insects (for which the reflected light has the following characteristics:** ***d*** **>15%, 80^o^<α<100^o^) of the shiny black, matt black and matt grey horizontal test surfaces used in experiments 1 and 2 measured with imaging polarimetry from two different directions of view in the blue (450 nm) part of the spectrum.** The polarimeter saw: (I) toward an open field (the surfaces reflected sun- and skylight), (II) toward trees and bushes (the surfaces reflected light from a tree canopy). The angle of elevation of the optical axis of the polarimeter was −45^o^ from the horizontal.
(DOC)

Figure S5 **Photograph, patterns of the degree of linear polarization** ***d*** **and the angle of polarization α (clockwise from the vertical), and areas detected as water by polarotactic insects (for which the reflected light has the following characteristics:** ***d*** **>15%, 80^o^<α<100^o^) of a shiny red car, the hood and roof of which are covered with matt black carbon foil.** The patterns were measured in the blue (450 nm) part of the spectrum with imaging polarimetry from two different directions of view under a cloudy sky when the sun was shining from behind a large thin cloud layer. The polarimeter saw toward the antisolar half of the sky. The angle of elevation of the optical axis of the polarimeter was −20^o^ from the horizontal. In the α-pattern double-headed arrows show the local direction of polarization of light reflected from the car-body. The number-plate of the car and two persons are screened by white rectangles.
(DOC)

Table S1 **Numbers of mayflies (M) and dolichopodids (D) landed on the shiny black, matt black and matt grey horizontal test surfaces in experiment 1 counted on the photographs taken after each permutation of the order of the surfaces.** No.: number of repetition of experiment, %: percentage of mayflies/dolichopodids relative to their total number counted on all three test surfaces, AV: average, SD: standard deviation. The number of repetition is 6 (see Materials and methods, and Discussion).
(DOC)

Table S2 **Numbers of three reactions (LA: landing, TO: touching, LO: looping) of tabanids to the shiny black, matt black and matt grey horizontal test surfaces in experiment 2 as a function of time in 2013 (6: June, 7: July).** The number of repetition is 20 (see Materials and methods, and Discussion).
(DOC)
