# Supplementary figures and images for: Unexpected Attraction of Polarotactic Water-Leaving Insects to Matt Black Car Surfaces: Mattness of Paintwork Cannot Eliminate the Polarized Light Pollution of Black Cars
Source: PLoS One. 2014 Jul 30;9(7):e103339. doi: 10.1371/journal.pone.0103339 (PMC4116178; doi:10.1371/journal.pone.0103339)

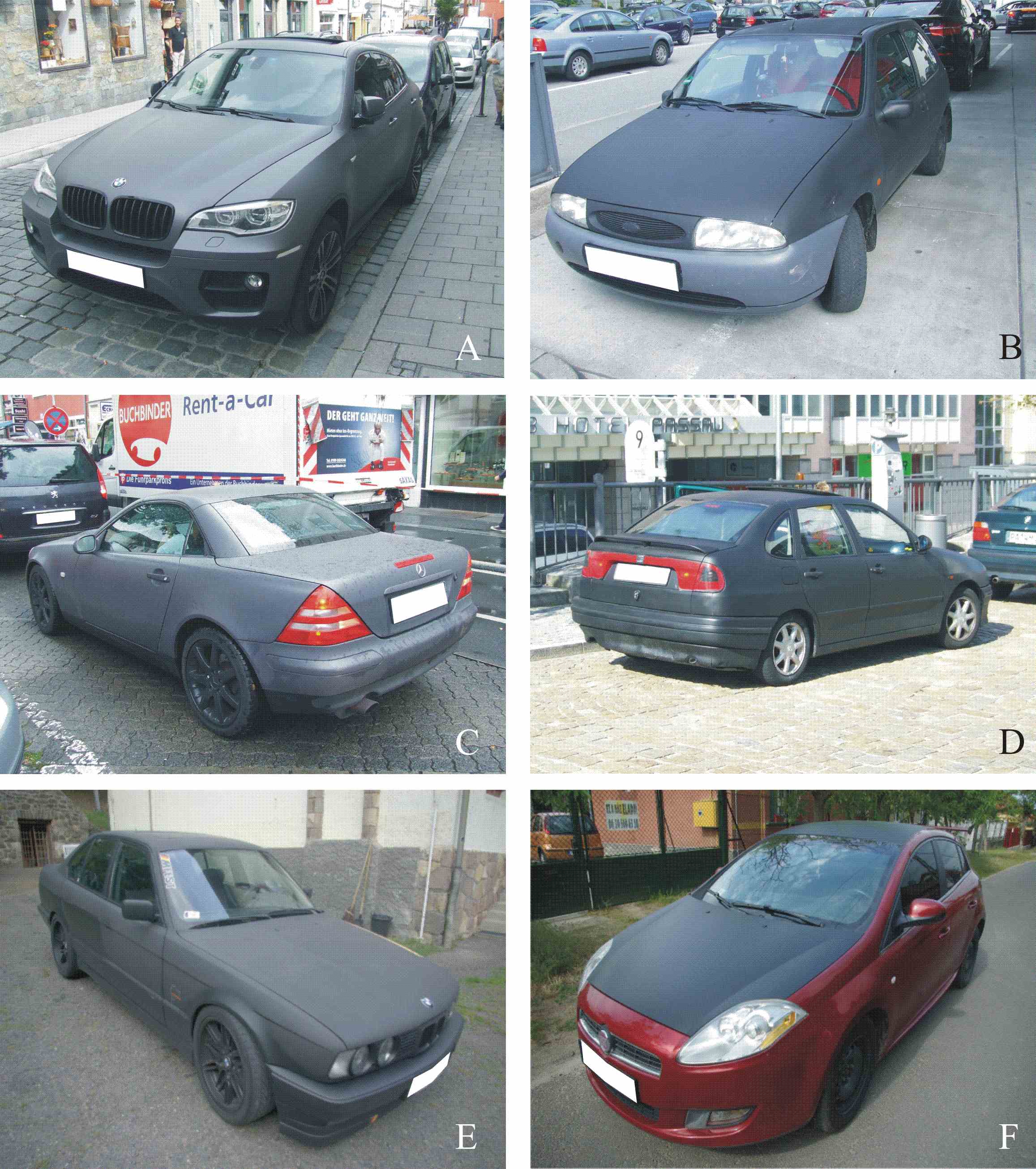


**Supplementary Figure S1**

Supplement: Figure S1 — Cars with different matt black/grey painting (A-E), or carbon foil on the hood and roof (F) (photographs taken by Gábor Horváth). The number-plates are screened by white rectangles. (DOC) [file pone.0103339.s001.doc]

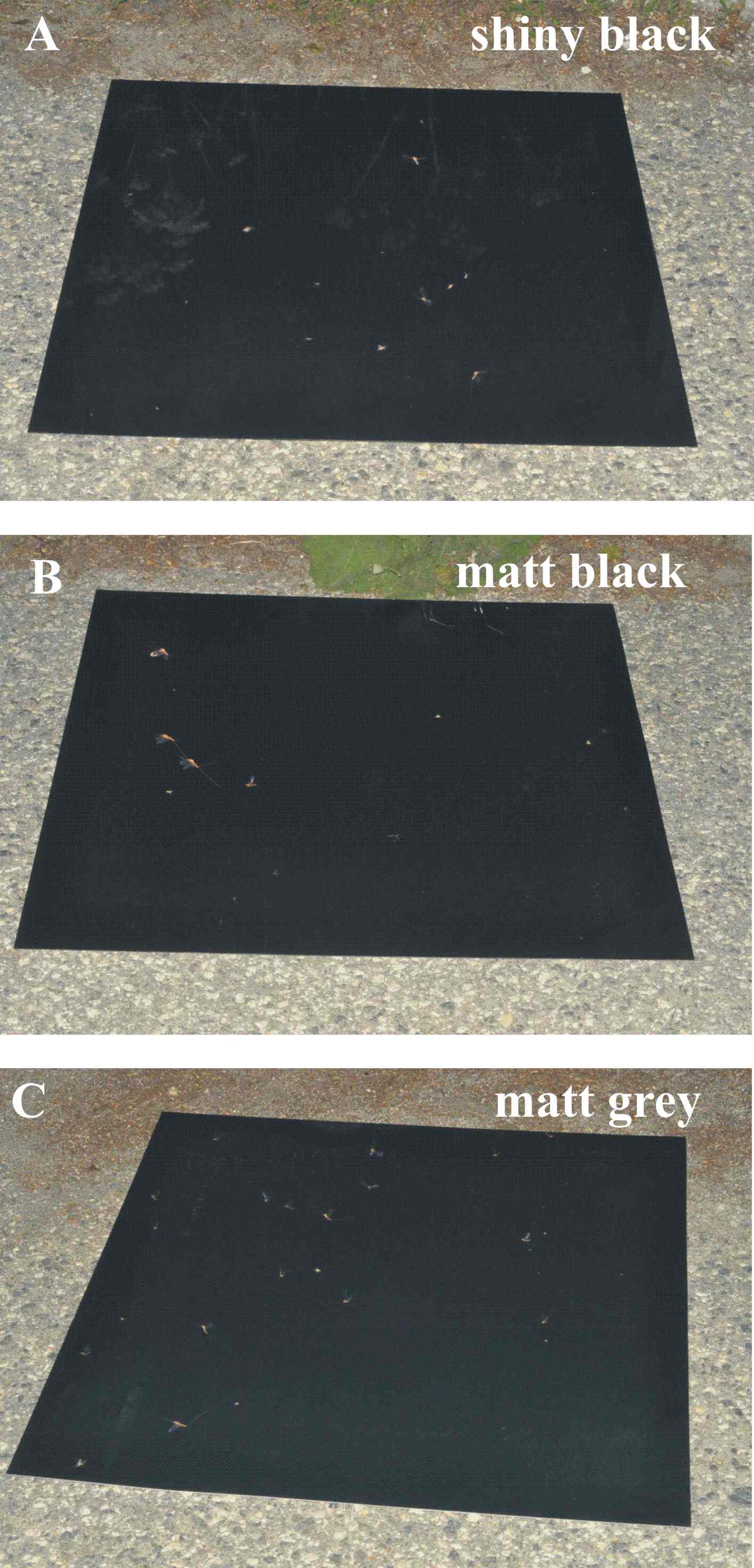


**Supplementary Figure S2**

Supplement: Figure S2 — Photographs of the shiny black, matt black and matt grey horizontal car-body fragments used in experiment 1 with some mayflies and dolichopodids above or on the test surfaces. On such photographs were counted the attracted insects. (DOC) [file pone.0103339.s002.doc]

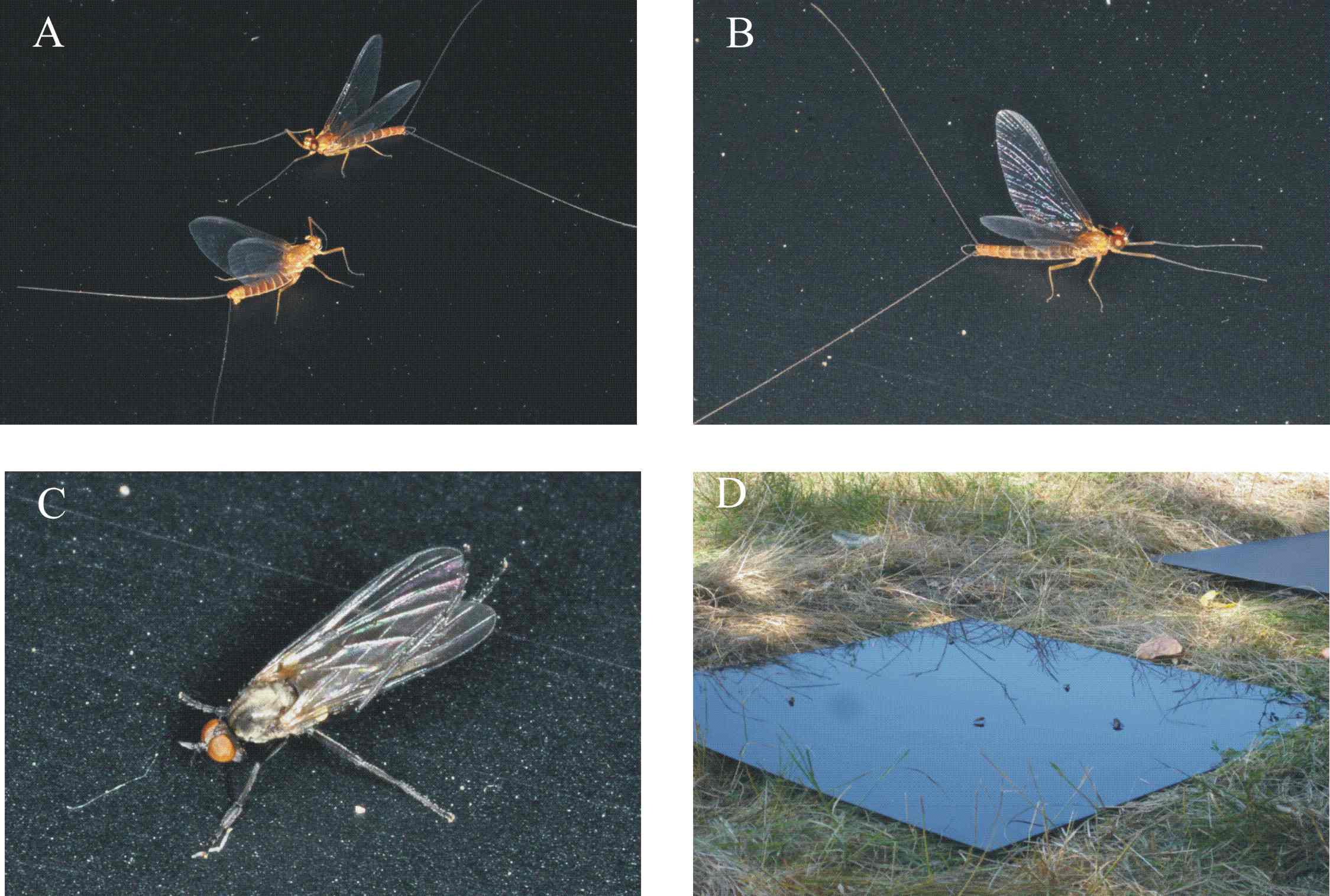


**Supplementary Figure S3**

Supplement: Figure S3 — Photographs of mayflies (A, B), dolichopodids (C) and tabanids (D) landed on the car-body fragments used in experiments 1 and 2. (A) An egg laying female (down) and a male (up) Rhithrogena semicolorata mayfly on the matt black test surface. (B) Male R. semicolorata on the shiny black test surface. (C) A dolichopodid fly on the matt black test surface. (D) Tabanid flies on the shiny black test surface. (DOC) [file pone.0103339.s003.doc]

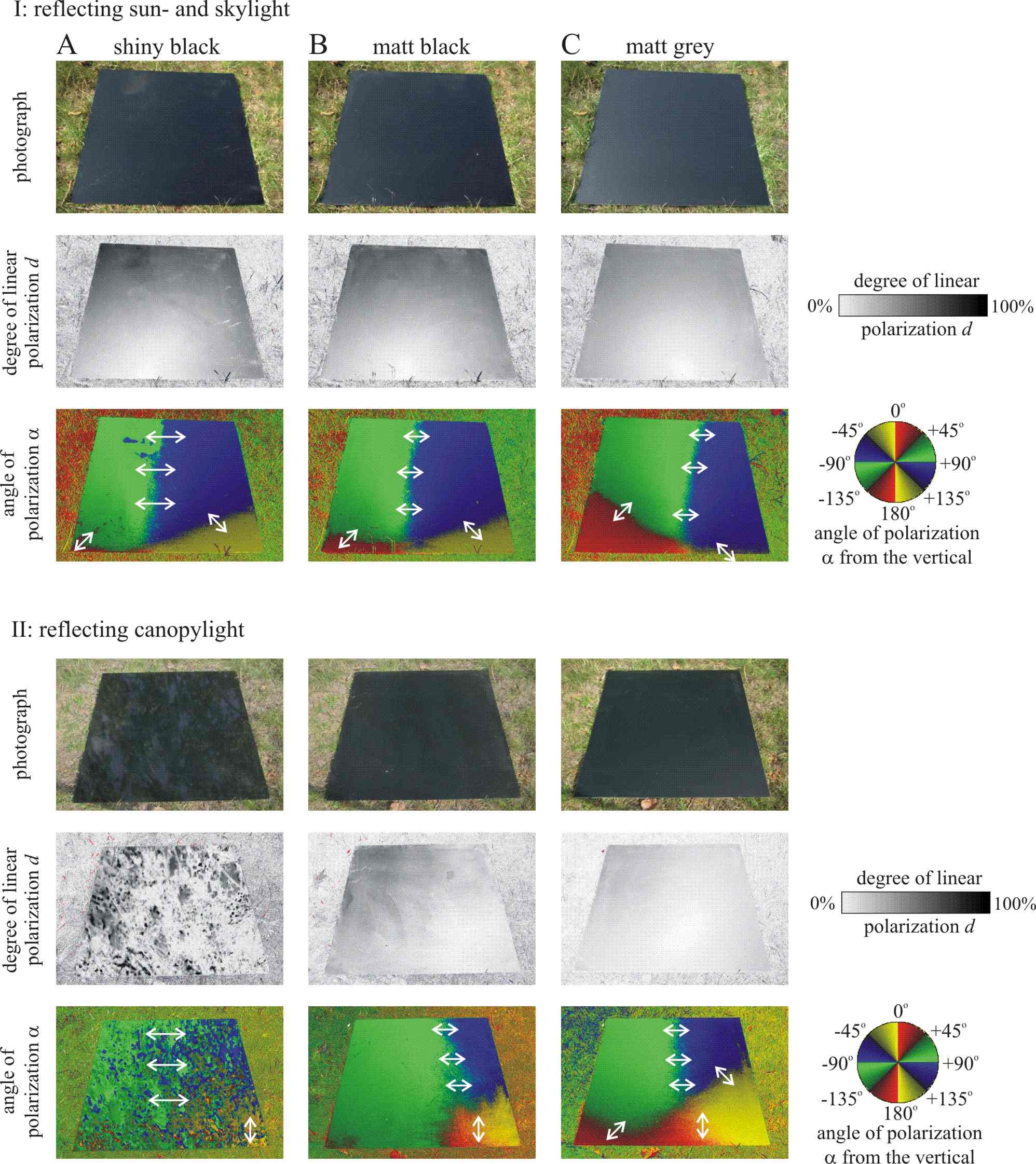


**Supplementary Figure S4**

Supplement: Figure S4 — Photograph, patterns of the degree of linear polarization d and the angle of polarization α (clockwise from the vertical), and areas detected as water by polarotactic insects (for which the reflected light has the following characteristics: d >15%, 80o<α<100o) of the shiny black, matt black and matt grey horizontal test surfaces used in experiments 1 and 2 measured with imaging polarimetry from two different directions of view in the blue (450 nm) part of the spectrum. The polarimeter saw: (I) toward an open field (the surfaces reflected sun- and skylight), (II) toward trees and bushes (the surfaces reflected light from a tree canopy). The angle of elevation of the optical axis of the polarimeter was −45o from the horizontal. (DOC) [file pone.0103339.s004.doc]

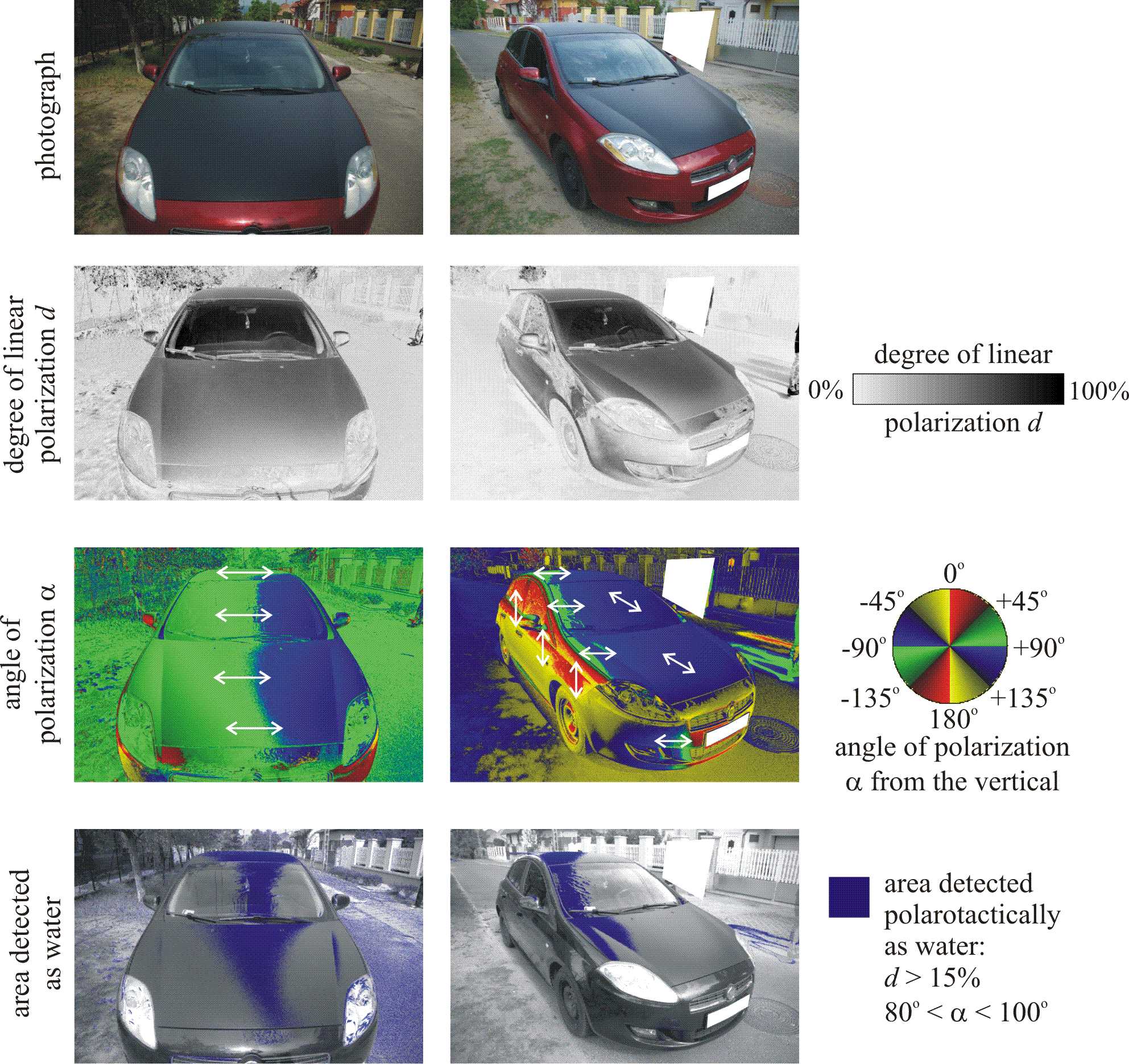


**Supplementary Figure S5**

Supplement: Figure S5 — Photograph, patterns of the degree of linear polarization d and the angle of polarization α (clockwise from the vertical), and areas detected as water by polarotactic insects (for which the reflected light has the following characteristics: d >15%, 80o<α<100o) of a shiny red car, the hood and roof of which are covered with matt black carbon foil. The patterns were measured in the blue (450 nm) part of the spectrum with imaging polarimetry from two different directions of view under a cloudy sky when the sun was shining from behind a large thin cloud layer. The polarimeter saw toward the antisolar half of the sky. The angle of elevation of the optical axis of the polarimeter was −20o from the horizontal. In the α-pattern double-headed arrows show the local direction of polarization of light reflected from the car-body. The number-plate of the car and two persons are screened by white rectangles. (DOC) [file pone.0103339.s005.doc]
