# Supplementary material for: Unexpected Attraction of Polarotactic Water-Leaving Insects to Matt Black Car Surfaces: Mattness of Paintwork Cannot Eliminate the Polarized Light Pollution of Black Cars
Source: PLoS One. 2014 Jul 30;9(7):e103339. doi: 10.1371/journal.pone.0103339 (PMC4116178; doi:10.1371/journal.pone.0103339)
Supplement: Table S1 — Numbers of mayflies (M) and dolichopodids (D) landed on the shiny black, matt black and matt grey horizontal test surfaces in experiment 1 counted on the photographs taken after each permutation of the order of the surfaces. No.: number of repetition of experiment, %: percentage of mayflies/dolichopodids relative to their total number counted on all three test surfaces, AV: average, SD: standard deviation. The number of repetition is 6 (see Materials and methods, and Discussion). (DOC) [file pone.0103339.s006.doc]

**Supplementary Table S1**

| **No.** | **shiny black** | | **matt black** | | **matt grey** | |
| --- | --- | --- | --- | --- | --- | --- |
|  | **M** | **D** | **M** | **D** | **M** | **D** |
| **1.** | 2 | 1 | 1 | 2 | 6 | 2 |
| **2.** | 3 | 1 | 1 | 2 | 9 | 2 |
| **3.** | 0 | 3 | 2 | 1 | 8 | 2 |
| **4.** | 0 | 5 | 0 | 2 | 6 | 3 |
| **5.** | 0 | 2 | 3 | 3 | 15 | 3 |
| **6.** | 0 | 1 | 0 | 5 | 13 | 2 |
| **7.** | 0 | 1 | 0 | 5 | 11 | 2 |
| **8.** | 0 | 3 | 1 | 0 | 8 | 3 |
| **9.** | 0 | 2 | 0 | 1 | 11 | 1 |
| **10.** | 1 | 6 | 0 | 0 | 8 | 1 |
| **11.** | 2 | 6 | 0 | 5 | 7 | 6 |
| **12.** | 3 | 2 | 1 | 8 | 3 | 0 |
| **13.** | 0 | 1 | 1 | 5 | 8 | 0 |
| **14.** | 0 | 0 | 0 | 3 | 10 | 2 |
| **15.** | 0 | 4 | 1 | 6 | 9 | 2 |
| **16.** | 0 | 1 | 1 | 4 | 16 | 2 |
| **17.** | 3 | 1 | 0 | 4 | 6 | 2 |
| **18.** | 3 | 0 | 4 | 4 | 11 | 2 |
| **19.** | 1 | 1 | 0 | 9 | 6 | 4 |
| **20.** | 0 | 2 | 3 | 2 | 13 | 2 |
| **21.** | 2 | 9 | 4 | 0 | 4 | 5 |
| **22.** | 0 | 6 | 2 | 8 | 5 | 4 |
| **23.** | 3 | 9 | 0 | 11 | 7 | 9 |
| **24.** | 4 | 11 | 1 | 6 | 8 | 16 |
| **25.** | 0 | 6 | 2 | 9 | 7 | 5 |
| **26.** | 0 | 6 | 4 | 4 | 18 | 4 |
| **27.** | 0 | 7 | 0 | 5 | 12 | 9 |
| **28.** | 0 | 10 | 0 | 8 | 17 | 4 |
| **29.** | 1 | 4 | 0 | 4 | 18 | 4 |
| **30.** | 1 | 5 | 0 | 7 | 8 | 9 |
| **31.** | 0 | 1 | 0 | 3 | 4 | 2 |
| **32.** | 0 | 1 | 0 | 2 | 3 | 2 |
| **33.** | 1 | 3 | 0 | 8 | 7 | 1 |
| **34.** | 1 | 0 | 0 | 3 | 5 | 1 |
| **35.** | 0 | 6 | 0 | 5 | 2 | 3 |
| **36.** | 0 | 12 | 0 | 5 | 1 | 1 |
| **37.** | 0 | 6 | 1 | 3 | 3 | 1 |
| **38.** | 0 | 4 | 0 | 4 | 2 | 1 |
| **39.** | 0 | 5 | 0 | 7 | 1 | 1 |
| **40.** | 0 | 6 | 0 | 6 | 0 | 0 |
| **41.** | 0 | 8 | 0 | 4 | 8 | 4 |
| **42.** | 0 | 6 | 0 | 3 | 6 | 2 |
| **43.** | 0 | 2 | 0 | 7 | 8 | 0 |
| **44.** | 0 | 6 | 0 | 6 | 5 | 4 |
| **45.** | 0 | 7 | 0 | 4 | 6 | 2 |
| **46.** | 0 | 6 | 0 | 5 | 4 | 2 |
| **47.** | 0 | 4 | 0 | 7 | 4 | 2 |
| **48.** | 0 | 2 | 0 | 3 | 5 | 2 |
| **49.** | 0 | 7 | 2 | 7 | 4 | 3 |
| **50.** | 0 | 5 | 2 | 3 | 2 | 1 |
| **51.** | 1 | 1 | 0 | 4 | 4 | 5 |
| **52.** | 0 | 5 | 1 | 3 | 5 | 3 |
| **53.** | 1 | 3 | 0 | 3 | 5 | 2 |
| **54.** | 1 | 3 | 1 | 0 | 7 | 1 |
| **55.** | 0 | 6 | 2 | 2 | 3 | 2 |
| **56.** | 1 | 4 | 0 | 1 | 4 | 2 |
| **57.** | 0 | 6 | 0 | 1 | 4 | 2 |
| **58.** | 0 | 5 | 1 | 0 | 4 | 3 |
| **59.** | 0 | 6 | 0 | 2 | 9 | 3 |
| **60.** | 1 | 4 | 0 | 4 | 7 | 3 |
| **61.** | 1 | 0 | 0 | 0 | 7 | 1 |
| **62.** | 0 | 3 | 3 | 5 | 15 | 2 |
| **63.** | 0 | 7 | 0 | 3 | 11 | 3 |
| **64.** | 0 | 7 | 0 | 4 | 11 | 4 |
| **65.** | 0 | 8 | 0 | 3 | 7 | 1 |
| **66.** | 0 | 1 | 0 | 1 | 16 | 2 |
| **67.** | 1 | 6 | 0 | 4 | 10 | 11 |
| **68.** | 2 | 4 | 1 | 2 | 6 | 5 |
| **69.** | 0 | 3 | 0 | 4 | 12 | 3 |
| **70.** | 1 | 5 | 0 | 8 | 6 | 3 |
| **71.** | 0 | 3 | 0 | 7 | 11 | 12 |
| **72.** | 1 | 7 | 0 | 4 | 8 | 5 |
| **73.** | 2 | 14 | 1 | 7 | 13 | 11 |
| **74.** | 1 | 10 | 1 | 8 | 12 | 9 |
| **75.** | 1 | 11 | 2 | 11 | 18 | 9 |
| **76.** | 0 | 8 | 0 | 6 | 14 | 7 |
| **77.** | 0 | 7 | 1 | 14 | 21 | 10 |
| **78.** | 0 | 8 | 2 | 8 | 13 | 5 |
| **79.** | 0 | 7 | 0 | 3 | 5 | 7 |
| **80.** | 0 | 4 | 0 | 2 | 8 | 4 |
| **81.** | 0 | 7 | 0 | 10 | 6 | 5 |
| **82.** | 0 | 6 | 0 | 5 | 6 | 2 |
| **83.** | 0 | 6 | 0 | 7 | 5 | 1 |
| **84.** | 0 | 6 | 0 | 6 | 7 | 4 |
| **85.** | 0 | 4 | 0 | 7 | 3 | 0 |
| **86.** | 0 | 6 | 0 | 6 | 3 | 2 |
| **87.** | 0 | 2 | 1 | 1 | 3 | 2 |
| **88.** | 0 | 4 | 2 | 4 | 4 | 0 |
| **89.** | 0 | 3 | 1 | 4 | 2 | 3 |
| **90.** | 0 | 5 | 0 | 5 | 6 | 2 |
| **91.** | 0 | 5 | 0 | 3 | 7 | 4 |
| **92.** | 0 | 6 | 1 | 2 | 5 | 4 |
| **93.** | 0 | 2 | 1 | 1 | 5 | 4 |
| **94.** | 0 | 4 | 1 | 0 | 5 | 2 |
| **95.** | 0 | 4 | 0 | 1 | 3 | 0 |
| **96.** | 2 | 14 | 0 | 1 | 6 | 4 |
| **sum** | **46** | **461** | **60** | **415** | **720** | **326** |
| **%** | **5.6** | **38.4** | **7.3** | **34.5** | **87.1** | **27.1** |
| **AV** | **0.48** | **4.80** | **0.63** | **4.32** | **7.50** | **3.40** |
| **SD** | **0.89** | **2.96** | **1.00** | **2.82** | **4.38** | **2.94** |
