# Supplementary material for: Unexpected Attraction of Polarotactic Water-Leaving Insects to Matt Black Car Surfaces: Mattness of Paintwork Cannot Eliminate the Polarized Light Pollution of Black Cars
Source: PLoS One. 2014 Jul 30;9(7):e103339. doi: 10.1371/journal.pone.0103339 (PMC4116178; doi:10.1371/journal.pone.0103339)
Supplement: Table S2 — Numbers of three reactions (LA: landing, TO: touching, LO: looping) of tabanids to the shiny black, matt black and matt grey horizontal test surfaces in experiment 2 as a function of time in 2013 (6: June, 7: July). The number of repetition is 20 (see Materials and methods, and Discussion). (DOC) [file pone.0103339.s007.doc]

**Supplementary Table S2**

| **date** | **shiny black** | | | **matt black** | | | **matt grey** | | |
| --- | --- | --- | --- | --- | --- | --- | --- | --- | --- |
|  | **LA** | **TO** | **LO** | **LA** | **TO** | **LO** | **LA** | **TO** | **LO** |
| 6.24 | 124 | 17 | 5 | 85 | 24 | 2 | 37 | 4 | 4 |
| 7.01 | 147 | 389 | 43 | 95 | 93 | 29 | 42 | 61 | 17 |
| 7.02 | 138 | 350 | 24 | 72 | 68 | 12 | 39 | 58 | 14 |
| 7.03 | 135 | 490 | 39 | 103 | 177 | 37 | 73 | 71 | 21 |
| 7.04 | 397 | 612 | 143 | 97 | 184 | 40 | 60 | 46 | 32 |
| 7.05 | 117 | 371 | 203 | 86 | 161 | 89 | 56 | 73 | 38 |
| 7.08 | 245 | 708 | 204 | 82 | 143 | 52 | 61 | 83 | 48 |
| 7.09 | 251 | 529 | 119 | 97 | 152 | 21 | 67 | 71 | 18 |
| 7.10 | 105 | 400 | 60 | 89 | 147 | 40 | 73 | 81 | 19 |
| 7.15 | 59 | 100 | 13 | 16 | 19 | 8 | 15 | 16 | 5 |
| 7.16 | 100 | 214 | 27 | 47 | 104 | 14 | 30 | 58 | 10 |
| 7.17 | 130 | 377 | 94 | 83 | 180 | 57 | 61 | 45 | 39 |
| 7.18 | 253 | 474 | 128 | 101 | 116 | 47 | 99 | 149 | 73 |
| 7.19 | 172 | 247 | 72 | 99 | 82 | 23 | 47 | 38 | 19 |
| 7.22 | 120 | 280 | 28 | 70 | 135 | 17 | 37 | 84 | 11 |
| 7.23 | 167 | 378 | 30 | 135 | 326 | 24 | 104 | 191 | 20 |
| 7.24 | 183 | 403 | 28 | 98 | 194 | 17 | 56 | 82 | 7 |
| 7.26 | 144 | 233 | 18 | 88 | 137 | 14 | 64 | 56 | 14 |
| 7.27 | 161 | 384 | 30 | 99 | 276 | 47 | 66 | 79 | 18 |
| 7.28 | 121 | 224 | 43 | 82 | 126 | 41 | 51 | 66 | 15 |
| **sum** | **3269** | **7180** | **1351** | **1724** | **2844** | **631** | **1138** | **1412** | **442** |
